# Association between exposure to blood heavy metal mixtures and overactive bladder risk among U.S. adults: a cross-sectional study

**DOI:** 10.3389/fpubh.2025.1597321

**Published:** 2025-06-04

**Authors:** Yanlin Zhu, Yameng Wu, Yang Wang, Hua Yang, Meisheng Zhang, Hengxing Zhu, Xiaoke Chen

**Affiliations:** ^1^Department of Urology, Guangdong Provincial People's Hospital, Zhuhai Hospital (Jinwan Central Hospital of Zhuhai), Zhuhai, China; ^2^Department of Urology, Fuyang Hospital of Anhui Medical University, Fuyang, China; ^3^Department of Urology, The Second Affiliated Hospital of Dalian Medical University, Dalian, China; ^4^Department of Urology, Renmin Hospital, Hubei University of Medicine, Shiyan, China

**Keywords:** heavy metals, mixture, co-exposure, overactive bladder, NHANES

## Abstract

**Background:**

Increasing evidence has demonstrated that exposure to environmental heavy metals harms human health. However, information regarding the impact of co-exposure to metal mixtures on the risk of overactive bladder (OAB) was limited. Our study aimed to explore the joint effects of blood heavy metal mixtures on OAB risk.

**Methods:**

Data for this study were obtained from four National Health and Nutrition Examination Survey cycles (2011–2018). The effects of single metals on OAB risk were explored using multivariate logistic regression. Additionally, we used weighted quantile sum (WQS), quantile-based g computation (qgcomp), and Bayesian kernel machine regression (BKMR) models to explore the combined effect of metal mixtures on OAB risk. Age-stratified subgroup analyses were conducted, and restricted cubic splines (RCS) were utilized to investigate the non-linear relationship between metals and OAB.

**Results:**

A total of 4,183 individuals aged 20–80 years were included for further study. Among them, 866 (20.7%) participants had OAB. OAB patients had significantly higher blood concentrations of cadmium (Cd) and lead and lower blood concentrations of selenium and manganese than those without OAB (all *p* < 0.05). In the single-metal analyses, Cd significantly increased OAB risk. In the mixed-exposure analyses, the WQS and BKMR models consistently revealed a significant positive association between co-exposure to heavy metal mixtures and OAB risk, identifying Cd as the main positive driver. The young/middle-aged group exhibited similar significant associations. In the metal mixtures, Cd was the top-weighted metal for the entire population and young/middle-aged individuals, whereas mercury (Hg) held the highest weight among older adult individuals. Furthermore, we observed an underlying interaction between Cd and Hg in the BKMR model. In the sensitivity analyses, the findings from the qgcomp model validated the toxic effect of blood metal mixtures on OAB. According to the RCS regression, we identified a positive linear dose–response relationship between Cd and OAB risk.

**Conclusion:**

Our study identified that co-exposure to heavy metal mixtures was significantly related to OAB risk. Further research prioritizing low-dose, real-world exposure to metal mixtures in vulnerable populations (e.g., older adult, high-risk occupations) is essential to translate our findings into preventive strategies and regulatory policies.

## 1 Introduction

Overactive bladder (OAB) is a common urologic disorder that affects individuals of both men and women (1, 2). In 2014, the internationally recognized definition of OAB was established by the International Continence Society, which described OAB as a condition characterized by urgency of urination, with or without urge incontinence, usually with frequent daytime urination and nocturia, but without confirmed urinary tract infection or other known pathological abnormality (3). The prevalence of OAB differs across regions or countries and may be influenced by factors such as ethnicity and lifestyle practices. OAB was reported to affect 17% of males and 30% of females in the United States (4). In February 2025, Zhang et al. (5) investigated the global prevalence of OAB and reported that the prevalence was 20%. Among males, the prevalence is 16.1%, while it is 21.9% among females (5). Individuals may experience a heavy burden from the syndrome, which could result in sleep disturbances, anxiety or depression, an unpleasant sexual life, and decreased participation in social and physical activities, thus significantly influencing their quality of life (6). In the United States, OAB contributes to annual healthcare expenses amounting to billions of dollars (7). Despite continuous research efforts, the complicated pathophysiological mechanisms responsible for the onset and progression of OAB are not well illuminated.

Heavy metals, commonly identified as environmental pollutants, seriously threaten human health. Heavy metals generally refer to metallic elements with a density equal to or exceeding 5 g/cm^3^ (8). As industrialization and urbanization progress, heavy metal pollution has become increasingly widespread. These pollutants find their way into the external environment via multiple channels, including pesticides, exhaust gases, and industrial wastewater (9). Consequently, people are exposed to heavy metals via multiple pathways, such as air, food, water, and dermal contact. Over time, heavy metals can accumulate in tissues and organs, particularly in the heart, urinary system, and nervous system (10). Accumulating in this way may lead to several health concerns like cardiovascular disease, urinary disorder, and neurotoxicity (11–13). To date, few studies have focused on the association between exposure to heavy metals and the risk of OAB. Gao et al. (14) investigated the association between exposure to single metal cadmium (Cd) and OAB risk in a U.S. population aged 40 years and older and found that blood Cd levels were weakly positively related to the risk of OAB. Given the coexistence of heavy metals in the environment, real-world exposure to heavy metals is complex, involving potential synergistic, antagonistic, additive, or other interactions among various heavy metals. Thus, exposures to individual heavy metals might not fully account for the pathogenesis of OAB. Exploration of the joint effects of co-exposure to heavy metal mixtures on the risk of developing OAB is essential. The health effects of heavy metal mixtures in the environment are complex due to the potential interactions between components. Two critical mechanisms underlie mixture toxicity: synergism (where combined effects exceed the sum of individual effects) and antagonism (where one component reduces the toxicity of another). For example, Cd and lead (Pb) may synergistically enhance oxidative stress, while selenium (Se) might antagonize mercury (Hg) toxicity by forming Hg-Se particles with low bioavailability (15, 16). Traditional regression models (e.g., linear regression) often fail to capture these interactions because they assume additive effects and independence between exposures, leading to biased effect estimates. Advanced statistical approaches such as Bayesian kernel machine regression (BKMR) and weighted quantile sum (WQS) regression have been developed to address these limitations. BKMR flexibly models non-linear exposure-response relationships and component interactions through kernel functions, enabling the detection of synergy or antagonism even in high-dimensional mixtures (17). In contrast, WQS regression quantifies the overall mixture effect by assigning weights to components under a pre-specified direction (e.g., all toxic or protective), making it robust to multicollinearity (18). However, WQS assumes unidirectional effects and may underestimate antagonistic interactions, whereas BKMR allows bidirectional effects. By integrating both models, we aimed to comprehensively assess both the joint mixture effect and potential interaction mechanisms, thereby advancing beyond traditional approaches. Emerging evidence suggests that co-exposure to Cd and Hg may induce synergistic toxicity through shared biological pathways. Both metals are known to disrupt redox homeostasis by generating reactive oxygen species and depleting glutathione, a critical antioxidant (19). Experimental studies have shown that combined exposure to Cd and Hg exacerbates oxidative damage in renal and hepatic tissues compared to individual exposures, likely due to their additive or supra-additive effects on mitochondrial dysfunction and lipid peroxidation (20). For example, Cd may impair Hg excretion by competing for metallothionein binding, while Hg can enhance Cd-induced inflammatory responses via NF-κB activation (19). In the context of bladder dysfunction, these metals may synergistically target urothelial cells through oxidative stress and sensory nerve sensitization, potentially amplifying the risk of OAB symptoms. Therefore, we hypothesized that the Cd-Hg mixture would exhibit synergistic effects on OAB pathogenesis.

In this study, we carried out a cross-sectional study based on U.S. Civilian data from the National Health and Nutrition Examination Survey (NHANES). After heavy metals enter the human body, they are first transported to various tissues and organs through the blood. The heavy metal concentration in the blood directly reflects the recent exposure and the current level of heavy metals in the circulation. However, heavy metals in urine are the part that is excreted through the kidneys after metabolism. They may be affected by short-term excretion rates, renal function, and hydration status (such as water intake), and fluctuate greatly, making it challenging to represent the overall load. In addition, some heavy metals (such as Pb and Cd) have a relatively long half-life in the blood, which can more stably reflect chronic exposure and accumulation in the body. However, the concentration of heavy metals in urine may only reflect exposure or acute excretion within a few days, and has a relatively low sensitivity to long-term cumulative exposure (21, 22). Therefore, blood heavy metal concentrations (versus urine) are optimal for this topic. We conducted a multivariate logistic regression to investigate the effects of a single metal on the risk of OAB. In addition, we employed WQS, quantile-based g computation (qgcomp), and BKMR models to explore the joint effects of heavy metal mixtures on the risk of OAB. Our study provided new epidemiological insights into the correlations between heavy metal exposure and the risk of OAB, aiding in the identification of hazardous factors associated with OAB.

## 2 Methods

### 2.1 Study design and population

NHANES is an ongoing, nationwide, and cross-sectional survey carried out biennially to assess the health and nutrition of U.S. Civilians who are not institutionalized. All NHANES programs received support from the National Center for Health Statistics Ethics Review Committee, and participants gave written consent upon enrollment. For more details about NHANES, visit https://wwwn.cdc.gov/nchs/nhanes/Default.aspx.

**Figure 1:**
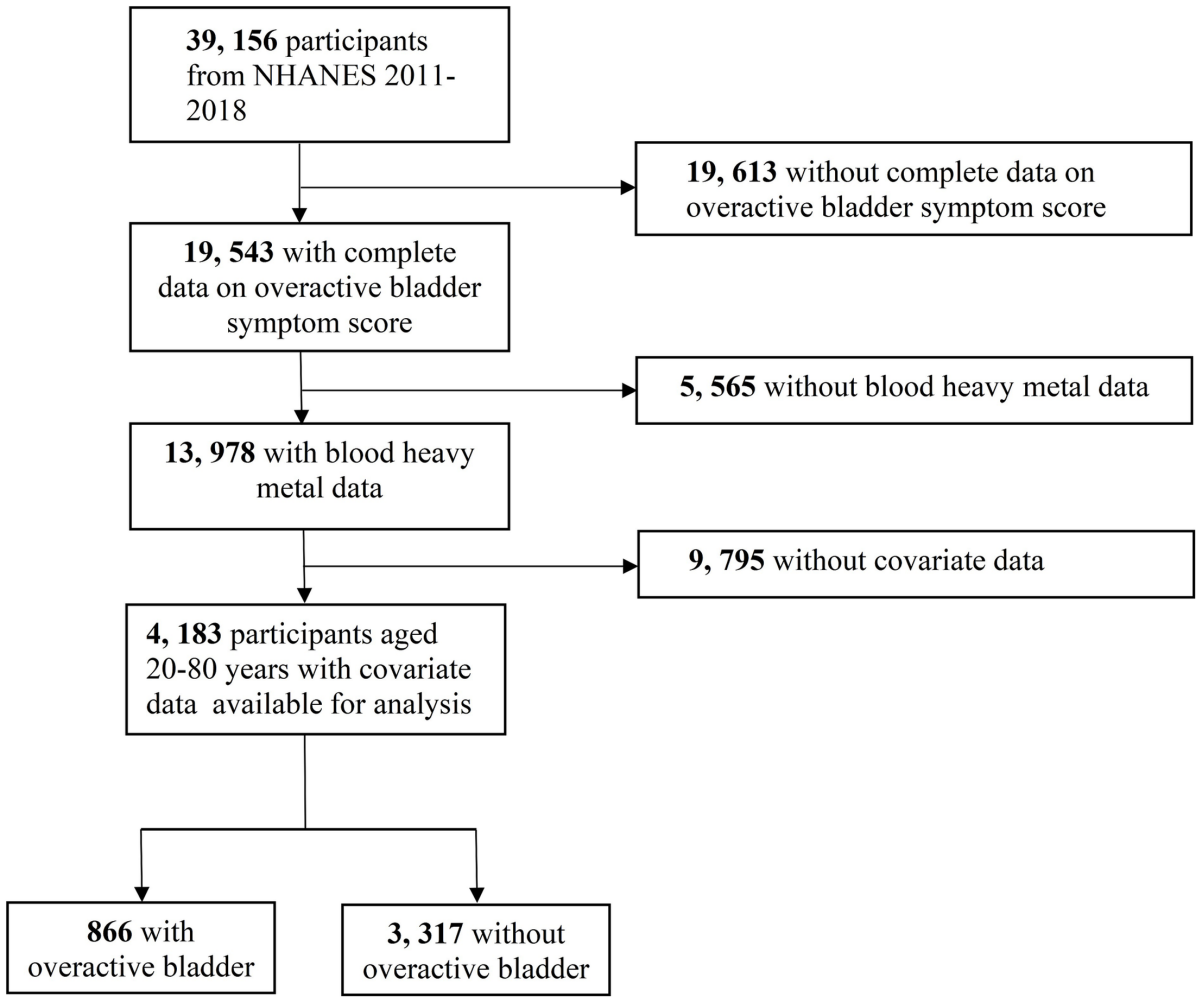
The flow diagram of selecting study population.

Data for this study were obtained from four NHANES cycles (2011–2012, 2013–2014, 2015–2016, 2017–2018). Participants were excluded if they did not provide complete data on overactive bladder symptom score (OABSS), lacked data on five blood heavy metals or covariates, or were under 20 years old. Finally, 4,183 participants were included; among them, 866 had OAB. The flow diagram of selecting the study population is presented in Figure 1.

### 2.2 Evaluation of symptoms associated with OAB

The main focus of this study is OAB. In this study, we employed the refined OABSS according to Blaivas et al. (23), together with the symptom frequency scoring method from the works of Song et al. (24) and Zhu et al. (25). This approach allows for integration with the NHANES database, transforming self-reported OAB symptoms into measurable metrics. OAB was featured as an overactive urination reflex, which manifested as urge urinary incontinence (UUI) and nocturia, as indicated by questionnaire responses. In-person interviews were conducted by trained personnel utilizing standardized questionnaires.

Two questions (KIQ044 and KIQ450) were used to evaluate the presence and severity of UUI: “During the past 12 months, {have you/has SP} leaked or lost control of even a small amount of urine with an urge or pressure to urinate and {you/he/she} could not get to the toilet fast enough?” “How frequently does this occur?” The assessment of nocturia burden was conducted through the question (KIQ480): “During the past 30 days, how many times per night did {you/SP} most typically get up to urinate, from the time {you/s/he} went to bed at night until the time {you/he/she} got up in the morning?” This validated assessment aligns with earlier research utilizing NHANES (26). A total score of three or higher suggests OAB. Related studies reported that the choice of a total score ≥ 3 cutoff as the diagnostic criterion of OAB achieved a sensitivity of 0.861 and specificity of 0.804, minimizing both false positives and false negatives in population-based studies (23, 27). Supplementary Figure S1 displayed the details of the scoring.

### 2.3 Heavy metals measurement

Four NHANES cycles (2011–2018) provided the detection results for five heavy metals in the blood. The concentrations of Cd, Hg, Pb, Se, and manganese (Mn) in whole blood were mainly determined using inductively coupled plasma mass spectrometry. The NHANES Lab Protocol web page provided detailed records of experimental methods and protocols. Following NHANES criteria, values under the limit of detection (LOD) were replaced by the LOD divided by the square root of two. LOD for Cd = 0.10 μg/L (8.5% < LOD), Hg = 0.28 μg/L (9% < LOD), Pb = 0.07 μg/dL (0.3% < LOD), Se = 24.48 μg/L (0% < LOD), and Mn = 0.99 μg/L (0% < LOD).

### 2.4 Covariates

In this study, we selected the following covariates based on the previously published literature (14, 28): age (20–59 years, ≥60 years), gender (female, male), race/ethnicity (Mexican American, Other Hispanic, Non-Hispanic White, Non-Hispanic Black, Other Race−Including Multi-Racial), education levels (below high school, high school/equivalent, collage and above), poverty income ratio (PIR) (<1.50, 1.50–3.50, >3.50), marital status (married/living with partner, widowed/divorced/separated, never married), body mass index (BMI) (<25, 25–29.9, ≥30), smoking status (never smoker, former smoker, current smoker), drinking status (non-drinker, drinker), hypertension (yes, no), diabetes mellitus (yes, no), and recreational physical activity (yes, no). Because NHANES often has incomplete records (missing OABSS, missing metal assays, etc.), we reported in Supplementary Table S1 the number and percentage of incomplete records for each variable.

### 2.5 Statistical analysis

Participants were classified based on the presence of OAB to compare baseline characteristics. All variables, except for five blood metal concentrations, were categorical and expressed as *n* (%), with comparisons made using chi-square tests. The concentrations of heavy metals in blood presented a non-normal distribution, were expressed as the median (Q1, Q3), and were compared using the Mann–Whitney U test. To acquire an approximate normal distribution, blood metal concentrations were transformed using the natural logarithm (Ln) and categorized into quartiles (Q1, Q2, Q3, Q4). We then employed Pearson correlation analysis to assess the correlations between the Ln-transformed blood metal concentrations. The categorization of Pearson’s correlation coefficients was as follows: weak (r ≤ 0.3), moderate (0.3 < r ≤ 0.8), and strong (r > 0.8). Age-stratified subgroup analysis was carried out, with groups defined as the young/middle-aged group (20 to 59 years) and the older adult group (60 years and above).

First, the effects of a single metal on the risk of OAB were explored using multivariate logistic regression. The first quartile (Q1) served as the reference group, and the results were expressed as odds ratios (ORs) along with their 95% confidence intervals (CIs). The models adjusted for underlying confounders, which included age, gender, race/ethnicity, education level, PIR, marital status, BMI, smoking status, drinking status, hypertension, diabetes mellitus, and recreational physical activity.

Second, we conducted WQS regression to evaluate the combined effect of five metal mixtures on the outcome because of its exceptional performance in characterizing environmental mixtures (18). The WQS analysis randomly divided the data into a training set (40%) and a validation set (60%). The weight of each metal was obtained by conducting 1,000 bootstrap iterations in the training set, followed by verifying the significance of the metal mixtures in the validation set. The R package (“gWQS”) was utilized to calculate the WQS index, consisting of weighted sums of individual metal concentrations. The WQS index, which ranged from 0 to 1, reflected mixed exposure levels of five blood heavy metals. The OR derived from the WQS model suggested the effect of a one-quartile increase in the co-exposure to the five heavy metal mixtures on the risk of OAB.

Moreover, due to the potential non-additive and non-linear dose–response relationships in mixture exposure, the BKMR model was utilized to evaluate the overall effect of five blood metals on the risk of OAB (17). This approach combined Bayesian techniques with statistical learning methods to assess nonlinearities and interactions in the exposure-outcome association. Characterized by its flexible modeling of exposure-response functions, BKMR facilitated the visualization of the effects of individual or joint exposures. The R package (“bkmr”) was employed to evaluate the combined effects of co-exposure to five blood metals on the risk of OAB following 25,000 iterations. The relative contribution of each component in the metal mixtures to the outcome was estimated by calculating the posterior inclusion probability (PIP) (29). In the meantime, we depicted bivariate exposure-response curves to visualize interactions between blood metals.

Additionally, we carried out several sensitivity analyses to ensure the robustness and reliability of the results. First, to adjust for the influence of other metals, multivariate logistic regression models incorporating all blood heavy metals were fitted. Second, we conducted the restricted cubic splines (RCS) regression using the R package (“rms”) to investigate the dose–response relationship between blood metal exposure and the risk of OAB further (30). The selection of knots, ranging from three to seven, was made to balance optimal fitting and overfitting of the principal spline, using the minimum absolute value of the Akaike information criterion as a guide. Ultimately, we selected three knots and utilized the median as a reference point. Finally, WQS regression can only evaluate exposures linked to outcomes in the same direction. Thus, the qgcomp model was introduced using the R package (“qgcomp”) to address this limitation. The qgcomp method allowed for the calculation of positive and negative weights for each factor of the mixtures without assuming homogeneity of directions (31).

The NHANES employs a complex multistage probability sampling design to represent the non-institutionalized U.S. population. To correct for oversampling of specific subgroups (e.g., racial/ethnic minorities) and non-response bias, NHANES provides statistical weights that integrate three components: (1) Base sampling weights: reflect the inverse probability of selection for each participant based on the survey’s stratified design; (2) Non-response adjustments: compensate for participants who did not complete specific examinations or questionnaires; (3) Post-stratification calibration: align the sample to U.S. Census population totals by age, sex, race/ethnicity, etc. The NHANES utilizes a complex multi-stage probability sampling method that generally needs sample weighting. However, previous research has indicated that weighted estimates may cause over-adjusted biases if the primary variables for calculating sample weights, such as gender, age, or race/ethnicity, are also used as covariates in the models (32–34). Thus, we used unweighted estimates to avoid this bias in this study. In addition, the following two factors were also the reasons why we conducted the unweighted analysis. (1) Focus on association, not prevalence: our research objective was to explore biological associations between heavy metal mixtures and OAB risk, rather than estimating population-level prevalence or attributable risk. Unweighted regression models are valid for testing exposure-outcome hypotheses when the sampling design is unrelated to the outcome. (2) Model complexity constraints: advanced mixture models (e.g., BKMR, WQS) require individual-level data and may not fully accommodate weighted likelihood estimation in standard software implementations. To ensure comparability across methods, we prioritized unweighted analyses. We utilized R version 4.2.2 and SPSS version 25.0 to perform all statistical analyses. Statistical significance was defined as a *p-*value less than 0.05.

## 3 Results

### 3.1 Population characteristics

**Figure 2:**
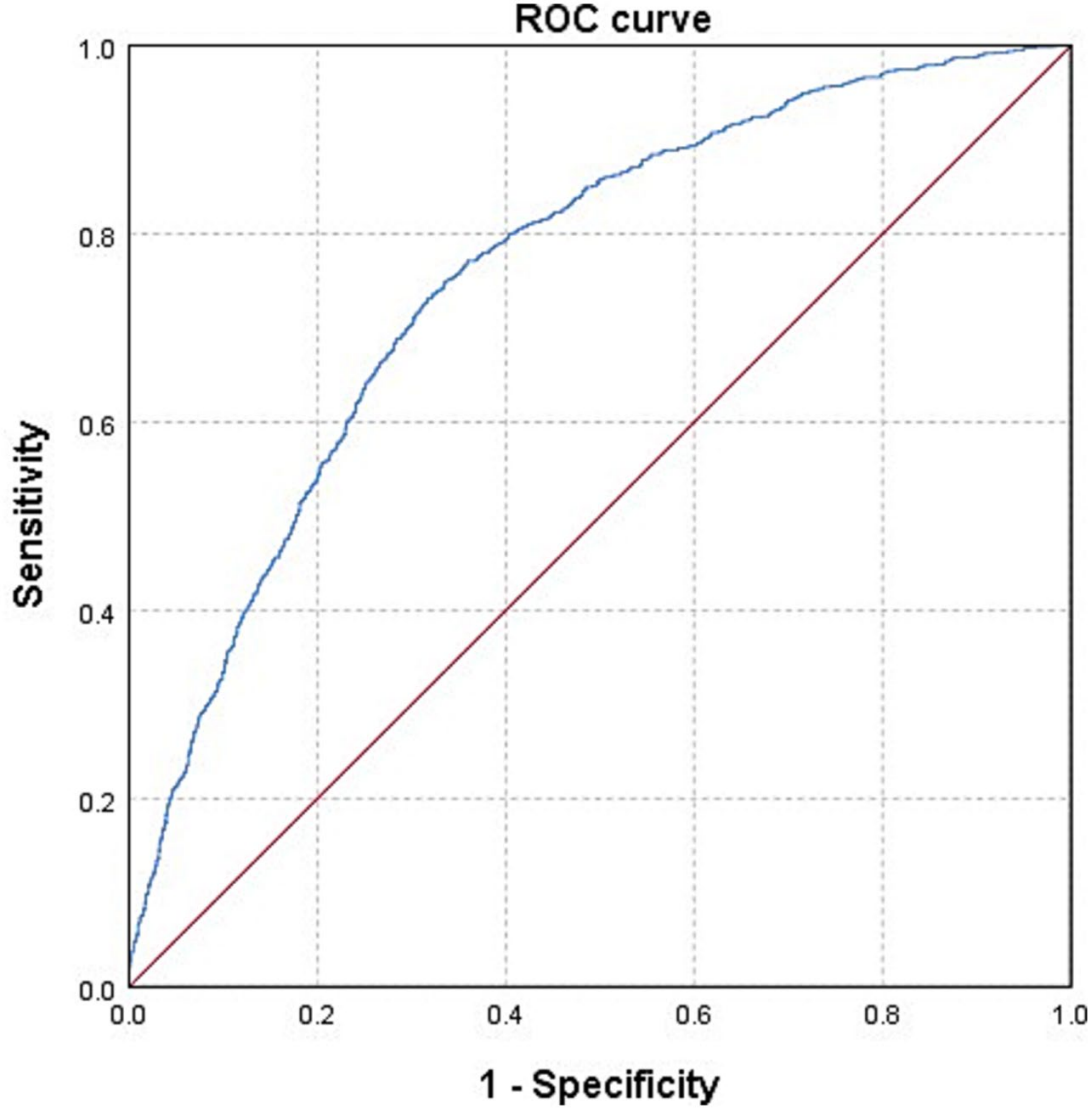
The ROC curve of evaluating the model’s ability to discriminate OAB cases from non-OAB cases. Model was adjusted for age, gender, race/ethnicity, education level, PIR, marital status, BMI, smoking status, drinking status, hypertension, diabetes mellitus, recreational physical activity, and five blood heavy metal concentrations.

The baseline characteristics of the 4,183 individuals from NHANES 2011–2018 were detailed in Table 1. Among them, 866 (20.7%) were diagnosed with OAB. Significant differences were observed in age, gender, race/ethnicity, education level, PIR, marital status, BMI, smoking status, drinking status, hypertension, diabetes mellitus, and recreational physical activity between the subgroups with and without OAB (all *p* < 0.001). Additionally, we depicted a receiver operating characteristic (ROC) curve and calculated the area under the ROC curve (AUC) to evaluate the model’s ability to discriminate OAB cases from non-OAB cases based on the included covariates. The ROC curve is presented in Figure 2. Moreover, the AUC value is 0.758 (95% CI 0.741–0.775).

**Table 1:** Baseline characteristics of participants in the NHANES 2011–2018 cycles.

Characteristics	Total	Non-overactive bladder	Overactive bladder	*p*-value
	(*n* = 4,183)	(*n* = 3,317)	(*n* = 866)	
Age group, *n* (%)				<0.001
20–59	2,848 (68.1)	2,491 (75.1)	357 (41.2)	
≥60	1,335 (31.9)	826 (24.9)	509 (58.8)	
Gender, *n* (%)				<0.001
Male	2,106 (50.3)	1752 (52.8)	354 (40.9)	
Female	2077 (49.7)	1,565 (47.2)	512 (59.1)	
Race/ethnicity, *n* (%)				<0.001
Mexican American	558 (13.3)	447 (13.5)	111 (12.8)	
Other Hispanic	449 (10.7)	352 (10.6)	97 (11.2)	
Non-Hispanic White	1,680 (40.2)	1,344 (40.5)	336 (38.8)	
Non-Hispanic Black	878 (21.0)	632 (19.1)	246 (28.4)	
Other Race—Including Multi-Racial	618 (14.8)	542 (16.3)	76 (8.8)	
Education level, *n* (%)				<0.001
Below high school	872 (20.8)	621 (18.7)	251 (29.0)	
High school/equivalent	906 (21.7)	704 (21.2)	202 (23.3)	
College and above	2,405 (57.5)	1992 (60.1)	413 (47.7)	
PIR, *n* (%)				<0.001
<1.50	1,578 (37.7)	1,183 (35.7)	395 (45.6)	
1.50–3.50	1,309 (31.3)	1,025 (30.9)	284 (32.8)	
>3.50	1,296 (31.0)	1,109 (33.4)	187 (21.6)	
Marital status, *n* (%)				<0.001
Married/ Living with Partner	2,457 (58.7)	1999 (60.3)	458 (52.9)	
Widowed/ Divorced/ Separated	865 (20.7)	573 (17.3)	292 (33.7)	
Never married	861 (20.6)	745 (22.5)	116 (13.4)	
BMI, *n* (%)				<0.001
<25	1,220 (29.2)	1,047 (31.6)	173 (20.0)	
25–29.9	1,361 (32.5)	1,105 (33.3)	256 (29.6)	
≥30	1,602 (38.3)	1,165 (35.1)	437 (50.5)	
Smoking status, *n* (%)				<0.001
Never smoker	2,366 (56.6)	1936 (58.4)	430 (49.7)	
Former smoker	1,011 (24.2)	759 (22.9)	252 (29.1)	
Current smoker	806 (19.3)	622 (18.8)	184 (21.2)	
Drinking status, *n* (%)				<0.001
Non-drinker	1,134 (27.1)	848 (25.6)	286 (33.0)	
Drinker	3,049 (72.9)	2,469 (74.4)	580 (67.0)	
Hypertension, *n* (%)				<0.001
No	2,444 (58.4)	2,133 (64.3)	311 (35.9)	
Yes	1,739 (41.6)	1,184 (35.7)	555 (64.1)	
Diabetes mellitus, *n* (%)				<0.001
No	3,623 (86.6)	2,955 (89.1)	668 (77.1)	
Yes	560 (13.4)	362 (10.9)	198 (22.9)	
Recreational physical activity, *n* (%)				<0.001
No	2,087 (49.9)	1,550 (46.7)	537 (62.0)	
Yes	2,096 (50.1)	1,767 (53.3)	329 (38.0)	
Blood heavy metals, median (Q1, Q3)				
Cd	0.300 (0.190, 0.560)	0.290 (0.180, 0.520)	0.370 (0.230, 0.690)	<0.001
Hg	0.790 (0.420, 1.600)	0.790 (0.410, 1.620)	0.790 (0.440,1.550)	0.882
Pb	1.000 (0.640, 1.590)	0.960 (0.620, 1.530)	1.200 (0.750, 1.923)	<0.001
Se	193.460 (179.230, 208.460)	193.920 (179.655, 208.560)	191.165 (177.350, 207.898)	0.027
Mn	9.280 (7.440, 11.610)	9.310 (7.510, 11.635)	9.140 (7.180, 11.550)	0.031

Categorical variables were presented as *n* (%); non-normally distributed continuous variables are expressed as median (Q1, Q3). NHANES, National Health and Nutrition Examination Survey; PIR, family Poverty Income Ratio; BMI, body mass index; *n*, numbers of participants; %, percentage; Q, quartile. Bold: *p* < 0.05.

### 3.2 Heavy metal concentrations and correlations

Supplementary Table S2 presented the distributions of five blood heavy metal concentrations. The detection rates of all blood metals were >90.0%. Both Se and Mn were detected in the blood of all participants, with Se exhibiting the highest concentration among the metals. OAB patients had significantly higher blood concentrations of Cd and Pb and lower blood concentrations of Se and Mn than those without OAB (all *p* < 0.05). The correlation between five heavy metals after Ln-transformation was assessed using Pearson correlation coefficients (Supplementary Figure S2). We found a moderate correlation between Cd and Pb (r = 0.33). There was a weak correlation between Hg and Pb (r = 0.15), Hg and Se (r = 0.14), Cd and Mn (r = 0.08), Hg and Mn (r = 0.06), Hg and Cd (r = 0.04). In addition, we unexpectedly observed a negative correlation between Pb and Mn (r = −0.08), Cd and Se (r = −0.06).

### 3.3 Multivariate logistic regression to evaluate the association between exposure to single metals and OAB risk

Multivariate logistic regression, adjusting for all covariates, was utilized to assess the association between individual heavy metals and the risk of OAB (Table 2). According to the results, Q2 (OR: 1.389; 95% CI: 1.075–1.798, *p* = 0.012), Q3 (OR: 1.387; 95% CI: 1.070–1.801, *p* = 0.014), and Q4 (OR: 1.442; 95% CI: 1.065–1.955, *p* = 0.018) of Cd were associated with a significantly higher risk of OAB than Q1. Consistently, each unit increase in Ln-Cd was associated with a 17.8% higher risk of OAB (*p* = 0.020). However, we found no significant associations between other heavy metals and OAB (all *p* > 0.05). Subsequently, we conducted an age-stratified subgroup analysis (Table 2). The results indicated that compared to Q1, the young/middle-aged group had an increased risk of OAB for Hg in Q2 (OR: 1.451; 95% CI: 1.061–1.990, *p* = 0.020) and Cd in Q2 (OR: 1.751; 95% CI: 1.220–2.539, *p* = 0.003), Q3 (OR: 1.734; 95% CI: 1.185–2.558, *p* = 0.005), and Q4 (OR: 2.094; 95% CI: 1.348–3.268, *p* = 0.001). Moreover, each unit increase in Ln-Cd was associated with a 37.5% higher risk of OAB in the young/middle-aged group (*p* < 0.001). Among the older adult group, no significant associations were observed between all metals and the risk of OAB.

**Table 2:** Association of blood heavy metals with overactive bladder and age subgroup, NHANES 2011–2018.

Blood metals	Q1	Q2		Q3		Q4		Continuous	
		OR (95% CI)	*p*-value	OR (95% CI)	*p*-value	OR (95% CI)	*p*-value	OR (95% CI)	*p*-value
Cd									
Overall	reference	**1.389 (1.075, 1.798)**	**0.012**	**1.387 (1.070, 1.801)**	**0.014**	**1.442 (1.065, 1.955)**	**0.018**	**1.178 (1.026, 1.353)**	**0.020**
20 ≤ Age < 60	reference	**1.751 (1.220, 2.539)**	**0.003**	**1.734 (1.185, 2.558)**	**0.005**	**2.094 (1.348, 3.268)**	**0.001**	**1.375 (1.152, 1.641)**	**<0.001**
Age ≥ 60	reference	1.161 (0.837, 1.612)	0.371	1.144 (0.826, 1.585)	0.419	1.215 (0.844, 1.748)	0.294	1.123 (0.925, 1.364)	0.242
Hg									
Overall	reference	1.224 (0.970, 1.545)	0.089	1.143 (0.900, 1.451)	0.273	1.272 (0.990, 1.636)	0.060	1.081 (0.986, 1.185)	0.098
20 ≤ Age < 60	reference	**1.451 (1.061, 1.990)**	**0.020**	1.199 (0.863, 1.669)	0.280	1.111 (0.781, 1.579)	0.556	1.049 (0.927, 1.185)	0.445
Age ≥ 60	reference	0.968 (0.697, 1.342)	0.844	1.221 (0.880, 1.696)	0.233	1.354 (0.968, 1.895)	0.077	1.081 (0.958, 1.220)	0.204
Pb									
Overall	reference	0.899 (0.696, 1.161)	0.414	0.965 (0.744, 1.253)	0.791	1.239 (0.950, 1.617)	0.114	1.144 (0.996, 1.312)	0.056
20 ≤ Age < 60	reference	1.050 (0.750, 1.471)	0.774	1.035 (0.738, 1.451)	0.842	1.298 (0.931, 1.815)	0.125	1.075 (0.898, 1.283)	0.430
Age ≥ 60	reference	0.824 (0.596, 1.138)	0.241	0.915 (0.659, 1.269)	0.594	1.207 (0.869, 1.678)	0.261	1.069 (0.879, 1.300)	0.501
Se									
Overall	reference	0.936 (0.745, 1.175)	0.569	0.893 (0.707, 1.128)	0.343	1.002 (0.796, 1.263)	0.984	0.922 (0.484, 1.756)	0.806
20 ≤ Age < 60	reference	0.982 (0.717, 1.346)	0.912	0.842 (0.606, 1.167)	0.304	0.941 (0.685, 1.292)	0.709	0.748 (0.292, 1.910)	0.544
Age ≥ 60	reference	0.839 (0.609, 1.155)	0.283	0.872 (0.629, 1.206)	0.407	0.881 (0.637, 1.217)	0.441	0.711 (0.302, 1.666)	0.434
Mn									
Overall	reference	0.815 (0.648, 1.025)	0.081	0.831 (0.657, 1.050)	0.121	0.959 (0.754, 1.220)	0.733	0.867 (0.674, 1.114)	0.265
20 ≤ Age < 60	reference	0.781 (0.562, 1.084)	0.140	0.940 (0.685, 1.290)	0.702	0.930 (0.676, 1.280)	0.657	0.869 (0.625, 1.207)	0.404
Age ≥ 60	reference	0.831 (0.602, 1.145)	0.258	0.835 (0.606, 1.151)	0.273	0.854 (0.618, 1.178)	0.335	0.777 (0.552, 1.091)	0.146

NHANES, National Health and Nutrition Examination Survey; OR, odds ratio; CI, confidence interval; Q, quartile; Continuous, Ln-transformed concentration of heavy metals in blood. The model was adjusted for age, gender, race/ethnicity, education level, PIR, marital status, BMI, smoking status, drinking status, hypertension, diabetes mellitus, and recreational physical activity. Bold: *p* < 0.05.

In the sensitivity analysis, multivariate logistic regression models incorporated all blood heavy metals to adjust for the confounding effects of other metals. The results showed that the Q2 (OR: 1.363; 95% CI: 1.053–1.768, *p* = 0.019), Q3 (OR: 1.352; 95% CI: 1.038–1.765, *p* = 0.026), and Q4 (OR: 1.407; 95% CI: 1.027–1.929, *p* = 0.034) of Cd significantly elevated the risk of OAB compared to Q1. Additionally, each unit increase in Ln-Cd was associated with a 16.3% higher risk of OAB (*p* = 0.040). In contrast, the Q2 (OR: 0.774; 95% CI: 0.613–0.976, *p* = 0.031) of Mn significantly lowered the risk of OAB compared to Q1 (Supplementary Table S3). Moreover, the results of the RCS analysis were presented in Supplementary Figure S3. The RCS analysis for overall validated linear dose–response relationships between the risk of OAB and the blood metals Cd, Hg, Pb, Se, and Mn (all *P* for nonlinear > 0.05, all *P* for overall < 0.05). The risk of OAB increased with increasing concentration of Cd.

### 3.4 WQS and qgcomp models to evaluate the associations between five metal mixtures and OAB risk

**Figure 3:**
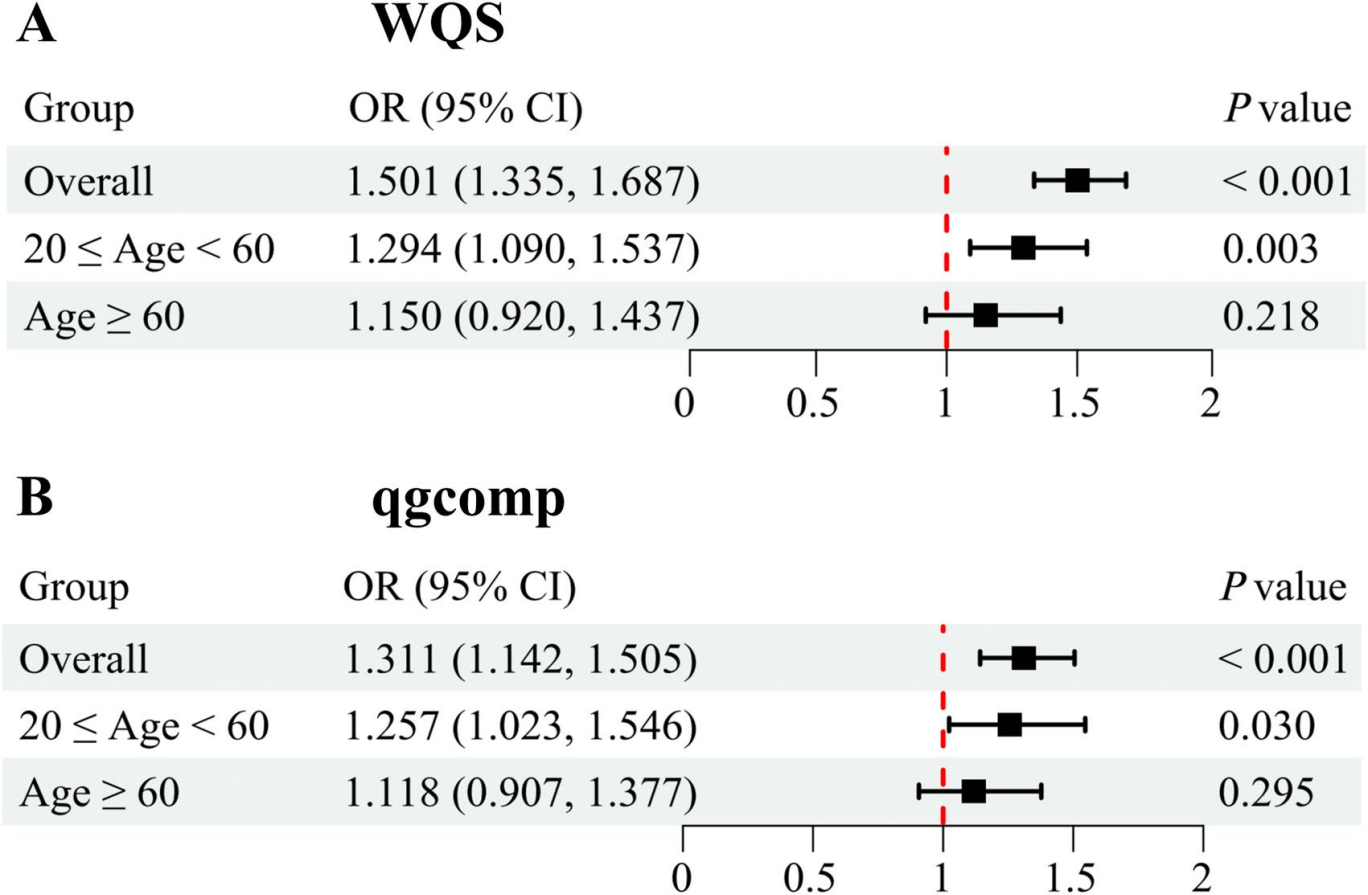
Odds ratios (95% CI) of overactive bladder associated with co-exposure to five blood metal mixtures by WQS **(A)** and qgcomp **(B)** analyses. Models were adjusted for age, gender, race/ethnicity, education level, PIR, marital status, BMI, smoking status, drinking status, hypertension, diabetes mellitus, and recreational physical activity.

**Figure 4:**
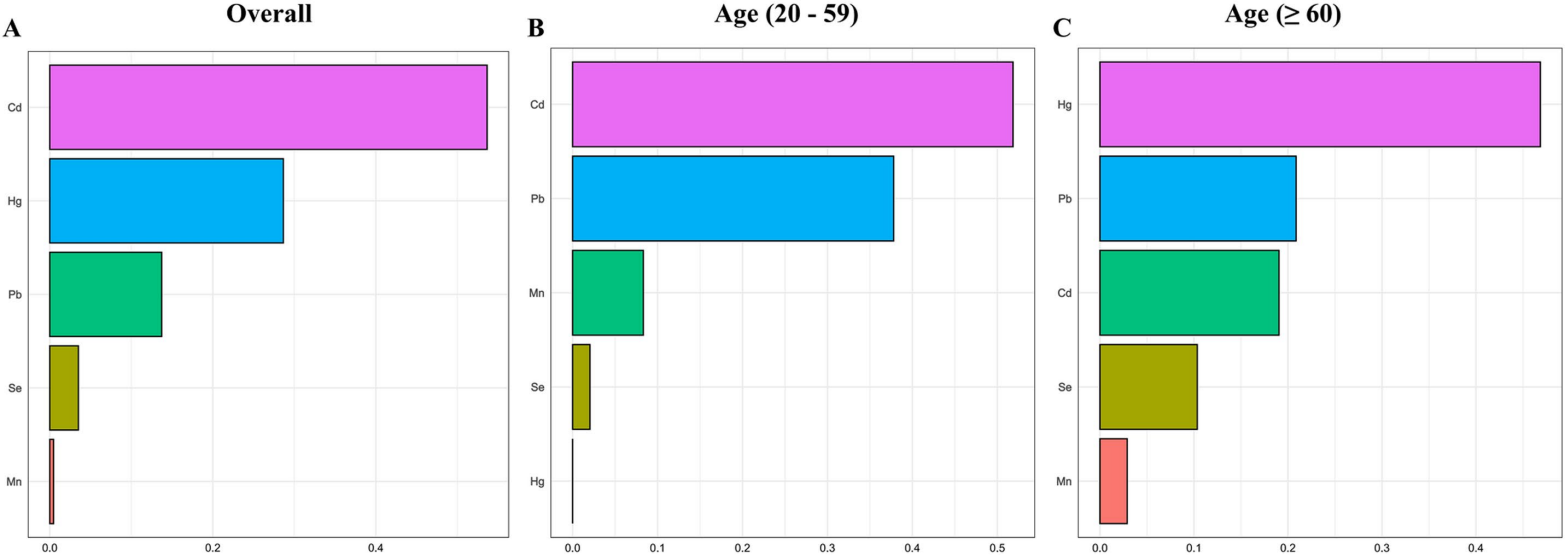
Weighted values of blood heavy metals for overactive bladder **(A)** and their subgroups **(B, C)** in the WQS model. The model was adjusted for age, gender, race/ethnicity, education level, PIR, marital status, BMI, smoking status, drinking status, hypertension, diabetes mellitus, and recreational physical activity.

After adjusting for all covariates, the finding from WQS index revealed a significant positive association between co-exposure to the five blood metal mixtures and the risk of OAB (OR: 1.501; 95% CI: 1.335–1.687, *p* < 0.001) (Figure 3A). In the age-stratified subgroup analysis, the WQS index suggested that the positive association between blood metal mixtures and OAB remained significant in the young/middle-aged group (OR: 1.294; 95% CI: 1.090–1.537, *p* = 0.003). However, the positive association was insignificant in the older adult group (OR: 1.150; 95% CI: 0.920–1.437, *p* = 0.218). Supplementary Table S4 detailed the estimated metal weights of OAB in the WQS model. Cd was the top-weighted metal for the entire population and young/middle-aged individuals, whereas Hg held the highest weight among older adult individuals (Figure 4).

**Figure 5:**
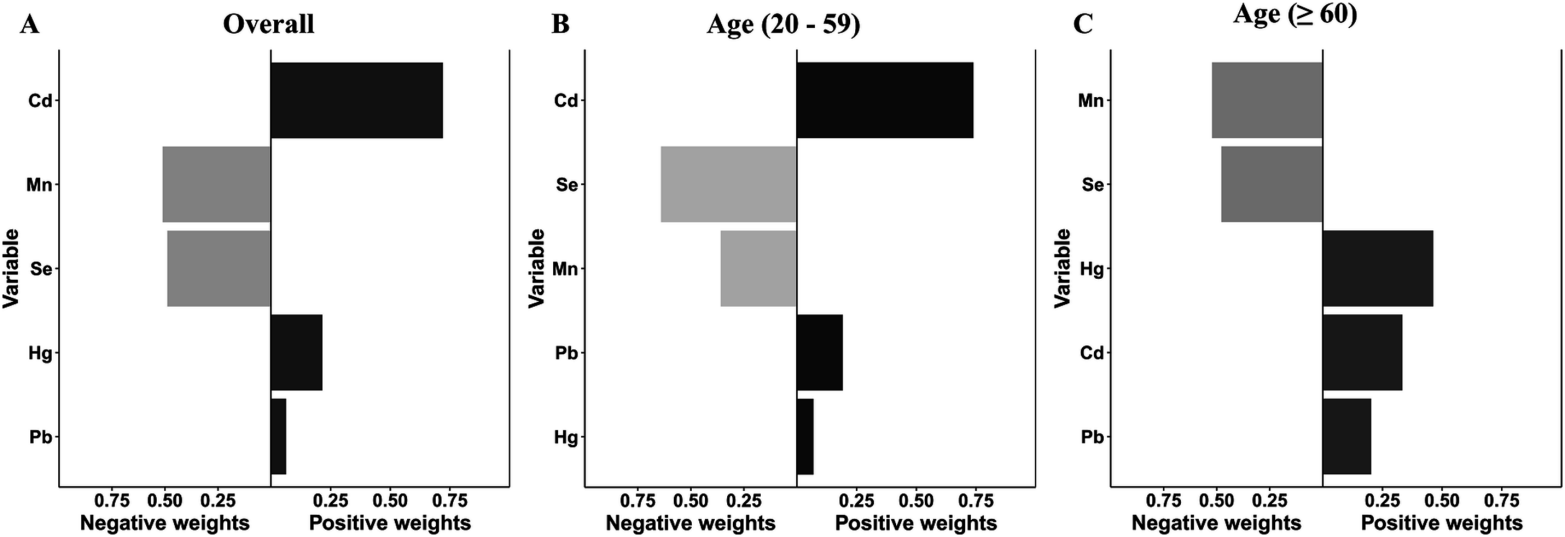
The weights of the positive and negative factors for overactive bladder **(A)** and their subgroups **(B, C)** in qgcomp model. The model was adjusted for age, gender, race/ethnicity, education level, PIR, marital status, BMI, smoking status, drinking status, hypertension, diabetes mellitus, and recreational physical activity.

Subsequently, the qgcomp model, which evaluated exposure weights in both positive and negative directions, was used for the sensitivity analysis. Similarly, our results identified a significant positive correlation between co-exposure to the five metal mixtures and the risk of OAB in the total population (OR: 1.311; 95% CI: 1.142–1.505, *p* < 0.001) and young/middle-aged group (OR: 1.257; 95% CI: 1.023–1.546, *p* = 0.030), but not in the older adult group (OR: 1.118; 95% CI: 0.907–1.377, *p* = 0.295) (Figure 3B). Supplementary Table S5 detailed the positive and negative metal weights of OAB in the qgcomp model. Cd, Hg, and Pb were positive drivers, while Se and Mn were negative drivers in the blood metal mixtures (Figure 5).

### 3.5 BKMR model to evaluate the associations between exposure to blood metal mixtures and OAB risk

**Figure 6:**
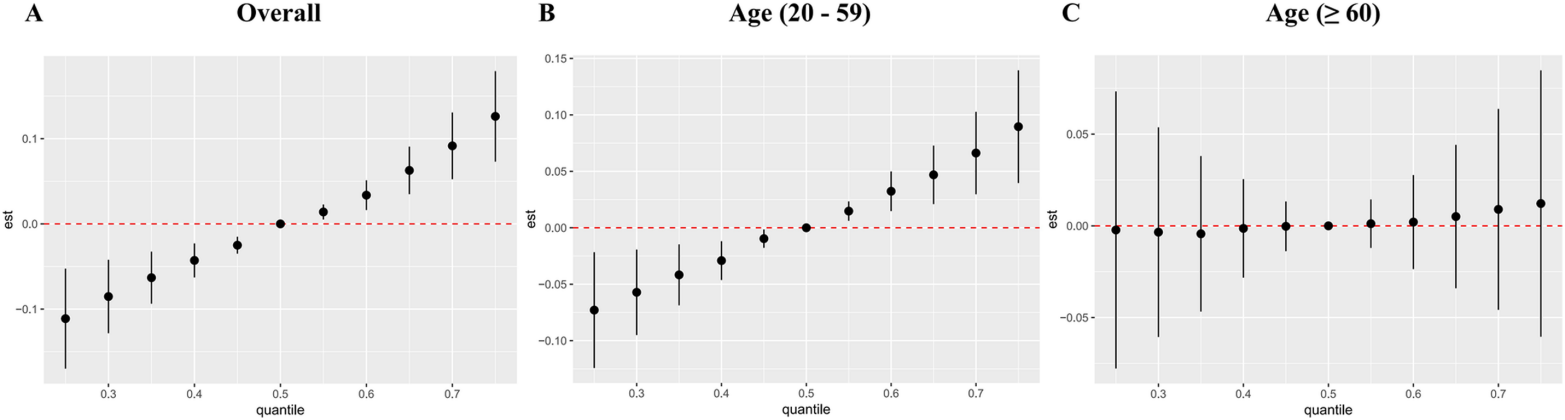
The joint effects of blood metal mixtures on the risk of overactive bladder were estimated by BKMR models in total population **(A)** and subgroups **(B, C)**. Models were adjusted for age, gender, race/ethnicity, education level, PIR, marital status, BMI, smoking status, drinking status, hypertension, diabetes mellitus, and recreational physical activity.

**Figure 7:**
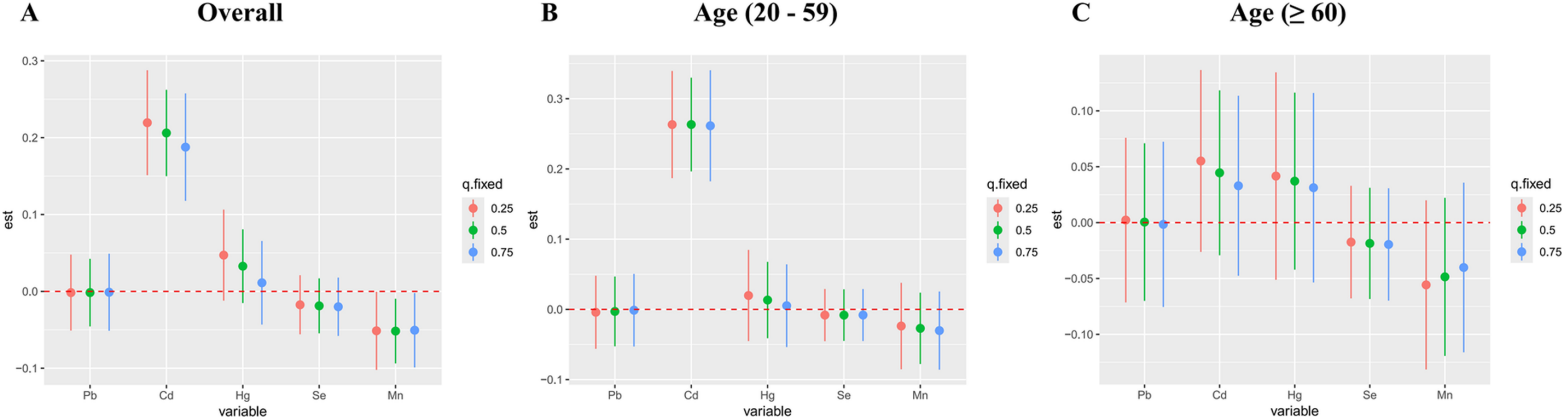
Associations of single blood metal with the risk of overactive bladder were estimated by BKMR models in total population **(A)** and subgroups **(B, C)**, when other all metals were held at their corresponding 25th (red), 50th (green), or 75th (blue) percentile, respectively. Models were adjusted for age, gender, race/ethnicity, education level, PIR, marital status, BMI, smoking status, drinking status, hypertension, diabetes mellitus, and recreational physical activity.

Figure 6 displayed the combined effect of five metal mixtures on OAB risk. For the entire population and young/middle-aged individuals, the risk of OAB was significantly increased when blood metal mixture concentrations were at or above the 55th percentile compared to the 50th percentile (Figures 6A,B). The older adult group also exhibited similar upward trends, although the correlations were insignificant (Figure 6C). Furthermore, with other blood metal concentrations maintained at the 25th, 50th, and 75th percentile, Cd significantly increased the risk of OAB in the entire population and young/middle-aged individuals (Figures 7A,B). The older adult group also showed similar positive effects, although the correlations were insignificant (Figure 7C). We also found that with other blood metal concentrations maintained at the 50th and 75th percentile, Mn significantly lowered the risk of OAB in the overall population (Figure 7A). The exposure-response function trend of five blood metals was presented in Supplementary Figure S4. With other heavy metal concentrations set at the median, we observed a positive association between blood Cd levels and the risk of OAB. In the overall population, the PIP values determined by the BKMR model for Cd, Se, and Mn were above the 0.5 threshold, whereas in the young/middle-aged group, only the PIP value for Cd exceeded 0.5 (Supplementary Table S6). The BKMR model also indicated that there may be underlying interactions between Cd and Hg (Supplementary Figure S5). To delve deeper into the statistical significance of this interaction, we employed a generalized linear model to examine the interaction. The results indicated a significant synergistic effect of Cd and Hg in increasing the risk of OAB (OR = 1.13; 95% CI 1.05–1.25, *p* = 0.021).

## 4 Discussion

As far as we know, this cross-sectional study is the first to explore the joint effects of co-exposure to blood heavy metal mixtures on the risk of OAB. Participants with OAB had significantly higher blood concentrations of Cd and Pb and lower concentrations of Se and Mn than those without the condition. Multivariate logistic regression models for single-metal and multi-metal exposures identified that exposure to Cd significantly increased OAB risk. At the same time, RCS analysis further validated the linear dose–response relationship between Cd and OAB risk. Furthermore, the WQS, qgcomp, and BKMR models consistently revealed a significant positive association between co-exposure to heavy metal mixtures and the risk of OAB. In the age-stratified subgroup analysis, we found a similar significant association among the young/middle-aged individuals but not in the older adult group. According to the WQS and BKMR model results, Cd was the main positive factors driving the overall effect. The qgcomp model results consistently validated the positive effects of heavy metal Cd.

Cd, one of the most hazardous metals, is extensively present in food, soil, industrial products, and water supplies. Long-term exposure to Cd can lead to serious health concerns, such as carcinogenicity, endocrine disruption, nephrotoxicity, and reproductive toxicity (35). Similar to the findings from Gao et al. (14), we identified a significant positive association between exposure to single metal Cd and the risk of OAB. There are multiple possible causes accounting for the pathophysiology of OAB, which are not entirely illuminated. According to Chu and colleagues (36), OAB can be categorized into idiopathic OAB, myogenic OAB (e.g., bladder outflow obstruction), neurogenic OAB (e.g., spinal cord injury), and inflammatory OAB (e.g., interstitial cystitis). Griffiths et al. (37) proposed that decreased central nervous system inhibition of the urination reflex may be a potential pathophysiological mechanism of OAB. Their findings showed that injury to the frontal cortex may disrupt bladder control. *In vitro* and *in vivo* studies have validated that Cd has harmful effects on the nervous systems, both peripheral (38) and central (39), which result in extensive clinical symptoms. The phenomenon of oxidative stress is due to an imbalance in the production and clearance of reactive oxygen species within cells. Prior research revealed that excessive oxidative stress, hypoxia, and loss of blood supply play a crucial role in OAB (40–42). Heavy metals like Cd, Hg, and Pb can trigger the production of reactive oxygen species, which depletes the antioxidant capacity of cells and causes oxidative stress (43, 44). Oxidative stress induced by heavy metals, indicated by abnormal expression of SOD, MDA, GSH, and 8-OHdG, can lead to inflammation and subsequent health issues (45). In addition, it may be regulated by multiple signaling pathways, such as the nuclear translocation of NF-κB, Keap1-Nrf2-ARE, and high expression of HIF. We speculate that these three heavy metals may be involved in the occurrence and progression of OAB by promoting oxidative stress.

Previous studies have indicated a relationship between inflammation and the onset and development of OAB. Long-term inflammation can alter bladder function and increase sensitivity, causing OAB symptoms to emerge (46, 47). Molecular imbalances in inflammatory proteins may cause the progression of OAB. Ma and colleagues (48) demonstrated that patients with OAB exhibited increased levels of inflammatory factors (e.g., TNF-*α*) and decreased levels of anti-inflammatory factors (e.g., IL-4). It is commonly accepted that either single or combined exposures to heavy metals, such as Cd, Pb, and Hg, can result in long-lasting inflammation (increased levels of TNF, IL-6, and IL-8), which may be involved in the development of OAB (49, 50). Chen et al. (51) conducted animal studies and reported that exposure to Cd could elevate IL-1β, COX2, iNOS, and HO-1 levels, activate the TNF-α/NF-κB signaling pathway, and trigger an inflammatory response. Furthermore, according to Taha et al. (52), the findings from animal experiments demonstrated that subacute Cd poisoning reduced detrusor muscle contractions and disrupted bladder function by blocking the Ca^2+^/calmodulin signaling pathway. These findings suggest that Cd affects bladder function via multiple mechanisms and pathways.

In addition to oxidative stress and inflammation, growing research indicates that heavy metals may increase the risk of OAB by altering the gut microbiota composition. Relevant studies have reported that alterations in the gut microbiota are related to OAB (53, 54). Okamoto et al. (54) reported that compared to non-OAB groups, individuals with OAB-daily urgency exhibited a significant decrease in levels of the genus *Bifidobacterium*, while levels of the genus *Faecalibacterium* were significantly increased. Increasing evidence from animal studies indicates that exposure to heavy metals disrupts gut microbiota homeostasis, causing inflammation (elevated levels of IL-1β and TNF), possibly through interference with protein synthesis (55, 56). According to a mouse model study, Cd exposure significantly reduced the abundance of gut microbiota and decreased the number of microorganisms that produce short-chain fatty acids (57). Another investigation conducted by Nielsen and colleagues (58) focused on the effects of exposure to Hg on the metabolic profiles and gut microbiota in mice and fish. They demonstrated that exposure to Hg disrupted gut microbiota homeostasis in both species, significantly reducing the relative abundance of several long-chain fatty acids, such as oleic acid and stearic acid. It is generally believed that oleic acid can improve insulin sensitivity, increase antioxidant capacity, and reduce levels of pro-inflammatory factors (59). In summary, despite the need for further exploration of the exact mechanisms involved, we have grounds to speculate that gut microbiota dysbiosis plays a crucial role in the impact of heavy metals on OAB.

Although previous studies have reported that exposure to single metal Cd is associated with an increased risk of OAB, there is a lack of information regarding the joint effects of metal mixtures. In this study, we employed newly established statistical methods to unveil the combined effects of heavy metal mixtures on OAB. Furthermore, increasing evidence indicates that the pathogenesis, clinical patterns, and prevalence of OAB differ between young/middle-aged individuals and the older adult (2, 46). Therefore, we carried out subgroup analyses by age. The findings from the WQS regression were essentially consistent with those from the BKMR model used in this research. We found a significant positive association between co-exposure to heavy metal mixtures and the risk of OAB, with Cd being primarily responsible for the overall effect. However, the findings from the subgroup analysis do not completely align with the overall results. A similar significant positive association was identified among the young/middle-aged individuals but not in the older adult group. Aging might be a primary factor contributing to this phenomenon. An essential feature of aging is the increase in oxidative stress (60), which suggests that older adult individuals have higher baseline levels of oxidative stress compared to young/middle-aged individuals. Previously, we introduced possible explanations for the association between heavy metals and OAB, beginning with an elevation in oxidative stress triggered by heavy metals. For the older adult group, the increased baseline levels of oxidative stress might mask the impact of oxidative stress elevation triggered by heavy metals, thus obscuring the association between exposure to heavy metal mixtures and OAB, consequently contributing to discrepancies in the subgroup analysis findings. Additionally, we found that Cd was the top-weighted metal for young/middle-aged individuals, whereas Hg held the highest weight among older adult individuals. Cd exposure in young/middle-aged individuals is primarily due to unhealthy lifestyle habits (e.g., smoking) and occupational exposures. When smoking, Cd enters the lungs with the smoke, and the absorption rate is as high as 50%. Young/middle-aged smokers may accumulate Cd due to long-term smoking. Some cheap whitening products may be illegally added to contain Cd compounds. Moreover, young people are more likely to experience occupational exposure to the heavy metal Cd, such as battery manufacturing (nickel-Cd batteries), electroplating industry, alloy production, pigment/dye processing, construction industry (welding Cd-containing metals or processing Cd-containing coatings). In contrast, Hg exposure in the older adult is mainly related to traditional medicines and dental materials. In some cultures, Hg-containing Chinese medicine or folk remedies (e.g., cinnabar) are used to calm the mind, detoxify the body, or treat chronic diseases. The older adult may be chronically exposed to inorganic Hg (e.g., Hg sulfide) due to reliance on traditional Chinese medicine. Moreover, amalgam was once widely used in dental fillings, and its elemental Hg may slowly release trace amounts of Hg vapor. The older adult may be chronically exposed to low doses of Hg from early use of amalgam fillings, especially decades ago. The detoxification function of the liver and kidneys is weakened in the older adult, which is more likely to lead to Hg accumulation. Interestingly, we observed an underlying interaction between Cd and Hg in the BKMR model. Although the exact biological mechanisms are poorly understood, they may be associated with oxidative stress and an imbalance in metal homeostasis (61). We also carried out sensitivity analyses, such as qgcomp and RCS models, to ensure the robustness and reliability of the findings. The qgcomp regression analysis consistently unveiled that blood metal mixtures significantly increased the OAB risk, identifying Cd as the main positive driver. According to RCS analysis, we identified a positive linear dose–response relationship between the blood metal Cd and the risk of OAB.

There are several advantages to our study. First, this study thoroughly evaluated the influence of single heavy metal exposure on the risk of OAB, bridging previous research gaps. Second, we employed several new and comprehensive statistical methods to explore the association between co-exposure to heavy metal mixtures and the risk of OAB in a large population from various perspectives. We obtained basically consistent results, which made us confident in their robustness and reliability. Nevertheless, we must acknowledge that our study has some limitations. First, recall bias could not be entirely avoided since we determined the diagnosis of OAB through a self-reported questionnaire. Second, we could not establish an inference of a causal relationship between co-exposure to heavy metal mixtures and OAB risk because of the cross-sectional nature of the study. Third, NHANES data are collected through mobile examination centers (MECs) following standardized protocols to minimize inter-center variability. However, specific MEC identifiers or operational details (e.g., technician training, equipment calibration) are not publicly available in NHANES datasets, precluding direct adjustment for field-center effects. Additionally, seasonal variation in blood metal concentrations (e.g., due to diet, air pollution, or sample stability) was not explicitly adjusted for in this study, as NHANES does not release detailed examination dates (only survey cycles). These are the inherent limitations of the NHANES study. Finally, blood metal concentration measurements were single-point and may not reflect long-term exposure. Future studies should consider serial measurements or hair-keratin analysis, which better capture metal accumulation over months. Additionally, OAB itself (e.g., frequent voiding) might alter dietary or physical activity patterns and thereby affect metal retention. OAB-related behaviors such as increased fluid intake, altered dietary habits (e.g., reduced consumption of metal-contaminated foods), or diuretic use may influence metal absorption, distribution, or excretion.

## 5 Conclusion

In summary, our study identified that exposure to single metal Cd significantly increased the risk of OAB. Furthermore, the mixed-exposure analyses consistently unveiled a significant positive association between co-exposure to blood heavy metal mixtures and the risk of OAB, identifying Cd as the main positive driver. The young/middle-aged group exhibited similar significant associations. Given the limitations of the NHANES study, well-designed prospective studies are required to confirm our findings in the future.

## Data Availability

Publicly available datasets were analyzed in this study. This data can be found at: https://wwwn.cdc.gov/nchs/nhanes/Default.aspx.
