# Supplementary material for: Association between exposure to blood heavy metal mixtures and overactive bladder risk among U.S. adults: a cross-sectional study
Source: Front Public Health. 2025 Jun 4;13:1597321. doi: 10.3389/fpubh.2025.1597321 (PMC12174091; doi:10.3389/fpubh.2025.1597321)
Supplement: Supplementary file 1 [file Supplementary_file_1.DOCX]

**Association between exposure to blood heavy metal mixtures and overactive bladder risk among U.S. adults: A cross-sectional study**

Yanlin Zhu^1^†, Yameng Wu^2^†, Yang Wang^1,3^, Hua Yang^4^, Meisheng Zhang^3^, Hengxing Zhu^3^, Xiaoke Chen^1^*
†These authors have contributed equally to this work and share first authorship

^1^Department of Urology, Guangdong Provincial People's Hospital, Zhuhai Hospital (Jinwan Central Hospital of Zhuhai), Zhuhai, China
^2^Department of Urology, Fuyang Hospital of Anhui Medical University, Fuyang, China
^3^Department of Urology, The Second Affiliated Hospital of Dalian Medical University, Dalian, China
^4^Department of Urology, Renmin Hospital, Hubei University of Medicine, Shiyan, China

*Correspondence: Xiaoke Chen, Department of Urology, Guangdong Provincial People's Hospital, Zhuhai Hospital (Jinwan Central Hospital of Zhuhai), No. 2, Hongyang Road, Jinwan District, Zhuhai, 519000, China

1. mail: 13903739007@163.com.


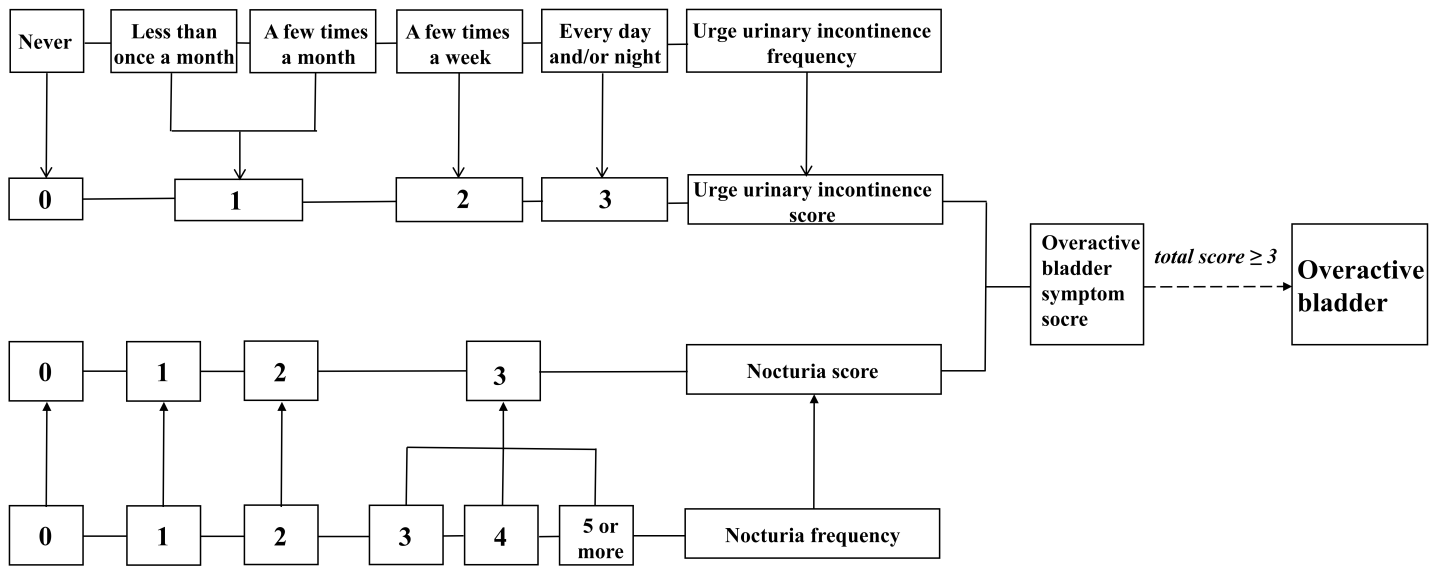


**Fig. S1** Flow diagram of the overactive bladder diagnosis based on overactive bladder symptom score.


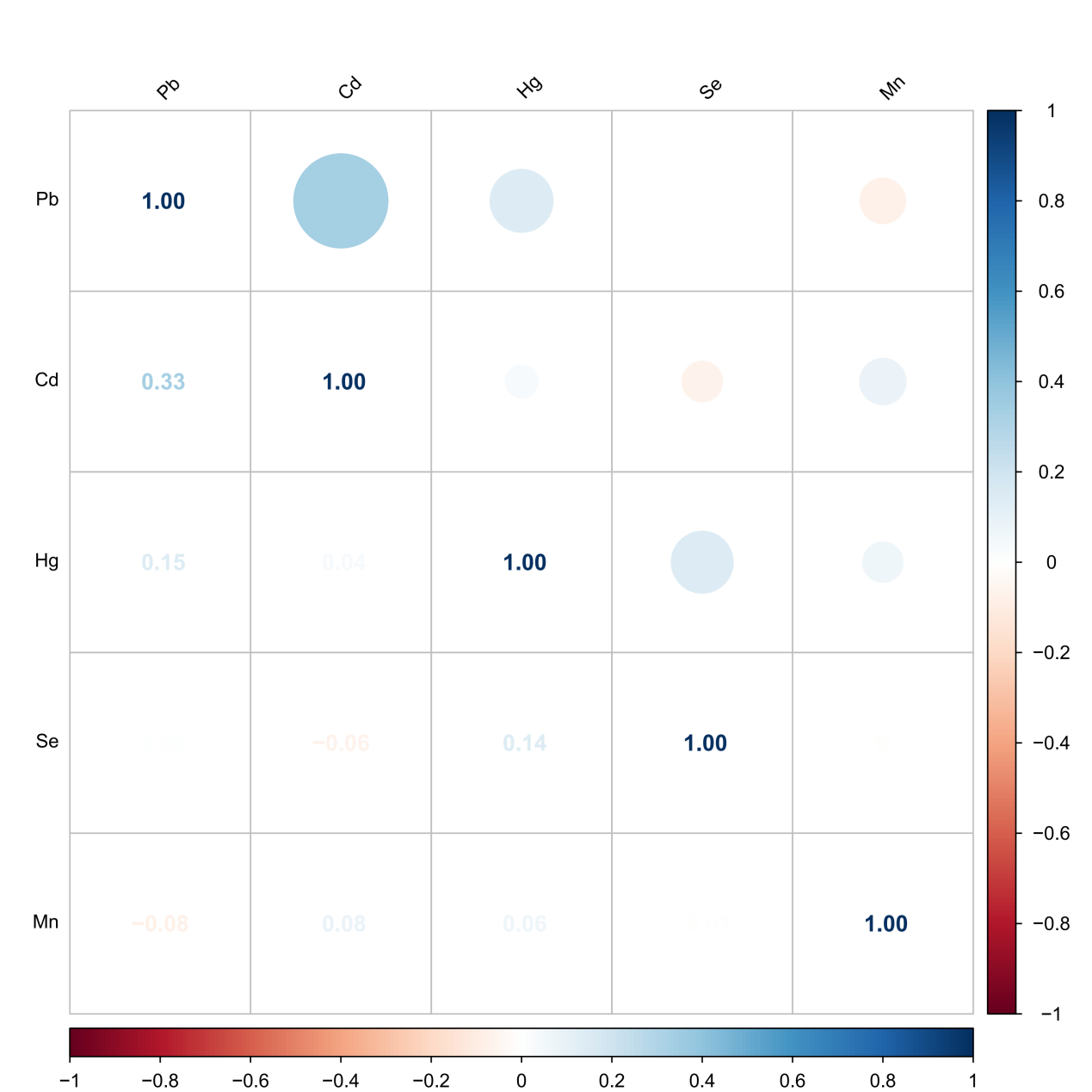


**Fig. S2** Pearson’s correlation matrix among Ln-transformed blood heavy metals.


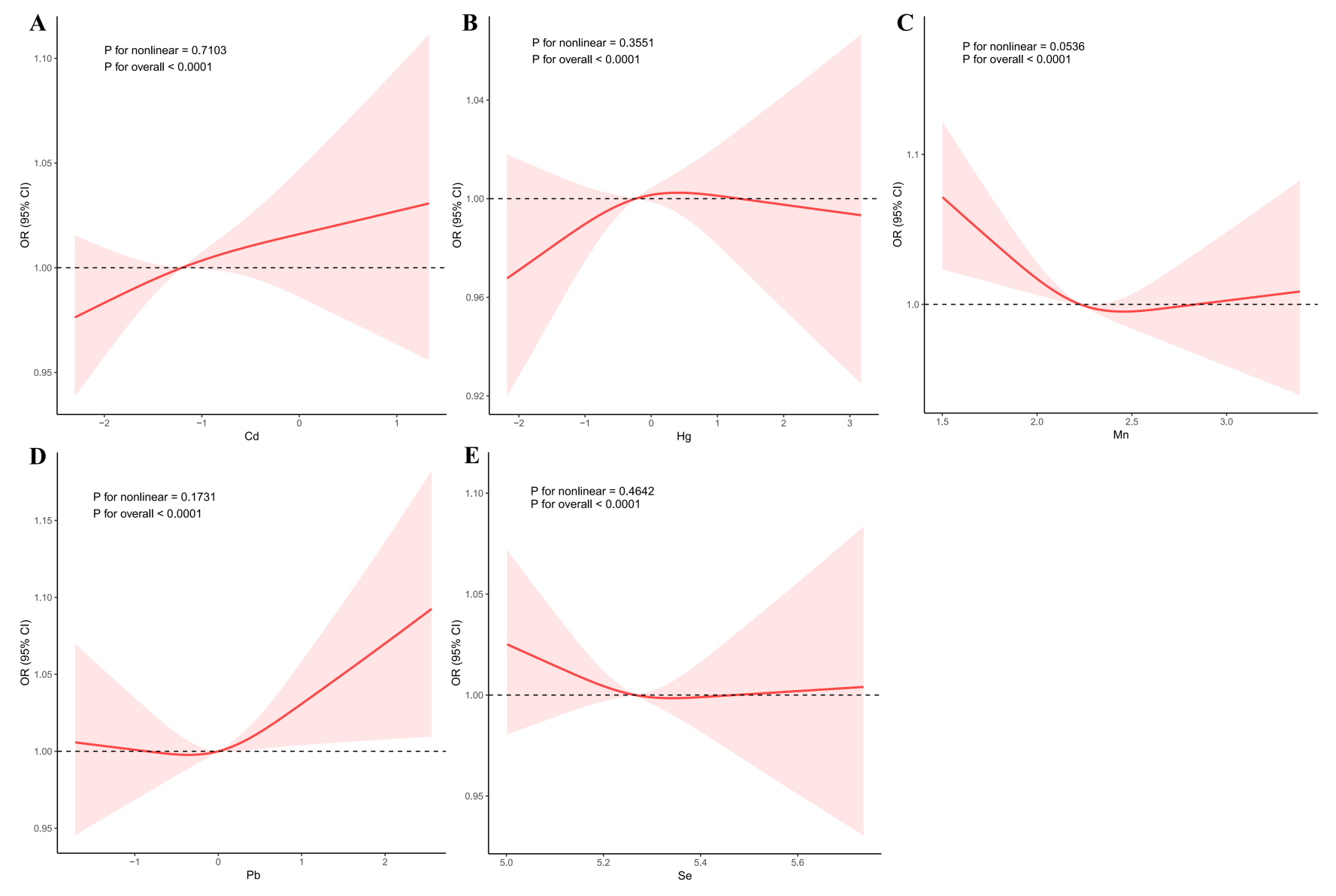


**Fig. S3** Restricted cubic spline plots of the association between Ln-transformed concentration of blood heavy metals and overactive bladder. The model was adjusted for age, gender, race/ethnicity, education level, PIR, marital status, BMI, smoking status, drinking status, hypertension, diabetes mellitus, and recreational physical activity.


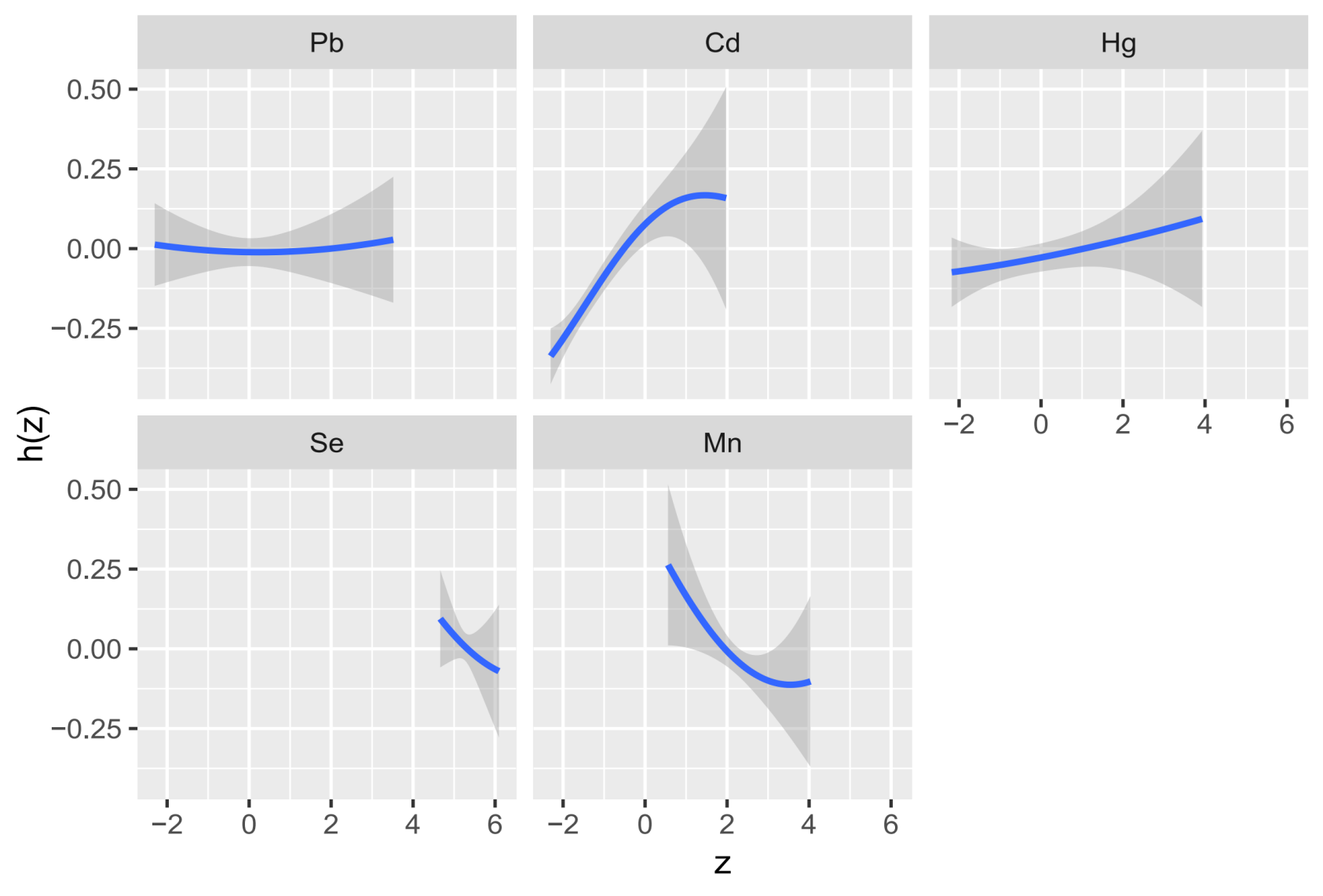


**Fig. S4** Univariate exposure-response function between each blood heavy metal and the prevalence of overactive bladder when the other heavy metals was fixed at 50th percentiles. The model adjusted for age, gender, race/ethnicity, education level, PIR, marital status, BMI, smoking status, drinking status, hypertension, diabetes mellitus, and recreational physical activity.


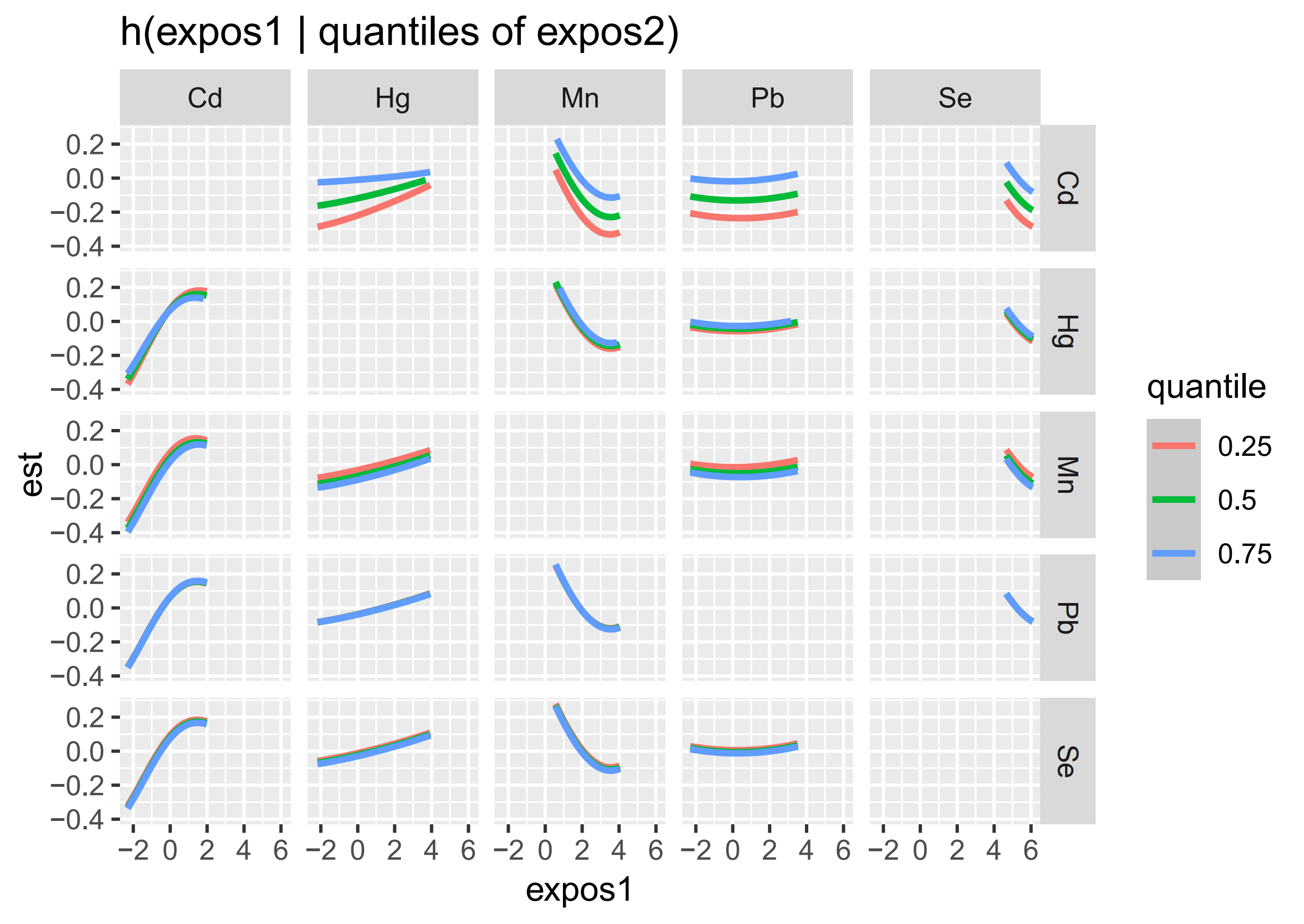


**Fig. S5** The interaction of blood heavy metals on overactive bladder. The model was adjusted for age, gender, race/ethnicity, education level, PIR, marital status, BMI, smoking status, drinking status, hypertension, diabetes mellitus, and recreational physical activity.

**Table S1** The number and percentage of missing records for each variable

| Variable | Total, n (%) | Missing, n (%) |
| --- | --- | --- |
| Age | 39,156 (100) | 0 (0) |
| Gender | 39,156 (100) | 0 (0) |
| Race/ethnicity | 39,156 (100) | 0 (0) |
| Education level | 39,156 (100) | 16,569 (42.3) |
| PIR | 39,156 (100) | 3,908 (10.0) |
| Marital status | 39,156 (100) | 16,558 (42.3) |
| BMI | 37,399 (100) | 2,981 (8.0) |
| Smoking status | 27,683 (100) | 4,184 (15.1) |
| Drinking status | 22,807 (100) | 7,215 (31.6) |
| Hypertension | 37,399 (100) | 7,775 (20.8) |
| Diabetes mellitus | 26,673 (100) | 1,148 (4.3) |
| Recreational physical activity | 33,702 (100) | 6,962 (20.7) |
| OABSS | 39,156 (100) | 19,613 (50.1) |
| Blood Cd | 29,069 (100) | 3,433 (11.8) |
| Blood Hg | 29,069 (100) | 3,433 (11.8) |
| Blood Pb | 29,069 (100) | 4,062 (14.0) |
| Blood Se | 29,069 (100) | 3,434 (11.8) |
| Blood Mn | 29,069 (100) | 3,434 (11.8) |

PIR, family Poverty Income Ratio; BMI, body mass index; OABSS, overactive bladder symptom score; Cd, cadmium; Hg, mercury; Pb, lead; Se, selenium; Mn, manganese; n, numbers of participants; %, percentage.

**Table S2** The distributions of 5 metals concentrations in blood in the NHANES 2011-2018 cycles

| Blood Metals | Detection  rates (%) | GM | Mean | SD | Min | P_25_ | P_50_ | P_75_ | Max |
| --- | --- | --- | --- | --- | --- | --- | --- | --- | --- |
| Cd | 91.5% | 0.338 | 0.486 | 0.543 | 0.110 | 0.190 | 0.300 | 0.560 | 7.230 |
| Hg | 91.0% | 0.864 | 1.553 | 2.670 | 0.113 | 0.420 | 0.790 | 1.600 | 50.810 |
| Pb | 99.7% | 1.032 | 1.349 | 1.453 | 0.177 | 0.640 | 1.000 | 1.590 | 33.670 |
| Se | 100% | 193.783 | 195.352 | 25.440 | 105.77 | 179.230 | 193.460 | 208.460 | 443.180 |
| Mn | 100% | 9.371 | 9.969 | 3.854 | 1.750 | 7.440 | 9.280 | 11.610 | 56.560 |

GM, geometric mean; SD, standard deviation.

**Table S3** Association of blood heavy metals with overactive bladder: multivariate logistic regression

| Blood metals | Q1 |  | Q2 |  |  | Q3 |  |  | Q4 |  |  | Continuous |  |
| --- | --- | --- | --- | --- | --- | --- | --- | --- | --- | --- | --- | --- | --- |
|  |  |  | OR (95% CI) | P value |  | OR (95% CI) | P value |  | OR (95% CI) | P value |  | OR (95% CI) | P value |
| Cd | Ref |  | **1.363  (1.053, 1.768)** | **0.019** |  | **1.352  (1.038, 1.765)** | **0.026** |  | **1.407  (1.027, 1.929)** | **0.034** |  | **1.163  (1.007, 1.343)** | **0.040** |
| Hg | Ref |  | 1.249  (0.989, 1.579) | 0.063 |  | 1.164  (0.914, 1.482) | 0.218 |  | 1.262  (0.977, 1.630) | 0.075 |  | 1.061  (0.966, 1.165) | 0.215 |
| Pb | Ref |  | 0.846  (0.651, 1.098) | 0.209 |  | 0.914  (0.700, 1.194) | 0.508 |  | 1.148  (0.871, 1.515) | 0.327 |  | 1.113  (0.964, 1.283) | 0.143 |
| Se | Ref |  | 0.937  (0.744, 1.180) | 0.580 |  | 0.912  (0.719, 1.156) | 0.448 |  | 1.022  (0.808, 1.293) | 0.854 |  | 0.889  (0.463, 1.703) | 0.724 |
| Mn | Ref |  | **0.774  (0.613, 0.976)** | **0.031** |  | 0.796  (0.628, 1.009) | 0.059 |  | 0.906  (0.709, 1.157) | 0.429 |  | 0.828  (0.643, 1.065) | 0.142 |

Models were adjusted for age, gender, race/ethnicity, education level, PIR, marital status, BMI, smoking status, drinking status, hypertension, diabetes mellitus, recreational physical activity, and five blood metals. Continuous, Ln-transformed concentration of variables. Ref, reference.

**Bold:** *P* < 0.05

**Table S4** The estimated metals weights of overactive bladder in WQS models

| Variables | Mean-weight | Variables | Mean-weight | Variables | Mean-weight |
| --- | --- | --- | --- | --- | --- |
| **Total** |  | **Age (20-59)** |  | **Age (≥60)** |  |
| Cd | 0.536 | Cd | 0.518 | Hg | 0.468 |
| Hg | 0.287 | Pb | 0.378 | Pb | 0.209 |
| Pb | 0.137 | Mn | 0.083 | Cd | 0.190 |
| Se | 0.035 | Se | 0.020 | Se | 0.103 |
| Mn | 0.005 | Hg | 0.000 | Mn | 0.029 |

**Table S5** The estimated metals weights of overactive bladder in qgcomp models

| Variables | Weight (P/N) | Variables | Weight (P/N) | Variables | Weight (P/N) |
| --- | --- | --- | --- | --- | --- |
| **Total** |  | **Age (20-59)** |  | **Age (≥60)** |  |
| Cd | 0.702 (P) | Cd | 0.739 (P) | Cd | 0.334 (P) |
| Hg | 0.225 (P) | Hg | 0.069 (P) | Hg | 0.463 (P) |
| Pb | 0.072 (P) | Pb | 0.192 (P) | Pb | 0.203 (P) |
| Se | 0.478 (N) | Se | 0.640 (N) | Se | 0.478 (N) |
| Mn | 0.522 (N) | Mn | 0.360 (N) | Mn | 0.522 (N) |

P, positive; N, negative.

**Table S6** The posteriori inclusion probability of single heavy metal in the NHANES 2011-2018 cycles

|  | PIP value | | |
| --- | --- | --- | --- |
| Blood metals | **Total** | **Age (20-59)** | **Age (≥60)** |
| Cd | 0.996 | 1.000 | 0.334 |
| Hg | 0.396 | 0.136 | 0.286 |
| Pb | 0.148 | 0.050 | 0.122 |
| Se | 0.510 | 0.192 | 0.378 |
| Mn | 0.694 | 0.230 | 0.386 |

PIP, posteriori inclusion probability.
